# Age-Old Dilemma of Pregnancy During Residency: A Cross-Sectional Study From Central India on Perceptions and Experiences of Female Medicos

**DOI:** 10.7759/cureus.38970

**Published:** 2023-05-13

**Authors:** Sarita K Sharma, Pragati G Rathod, Ujwala U Ukey, Sonali S Patil

**Affiliations:** 1 Community Medicine, Government Medical College & Hospital, Nagpur, IND

**Keywords:** experiences, perceptions, female, pregnancy, residency

## Abstract

Introduction

The integration of family and career poses a significant problem for women in the medical profession. Balancing residency program demands with burgeoning family issues has always been a dilemma for female medicos. Lack of support and sometimes hostility from life partners, program administrators, teachers, and other residents have been reported. The present study is an attempt to assess perceptions and experiences of female medicos regarding pregnancy during residency.

Methods

The present descriptive cross-sectional study was carried out in a government medical college and hospital, which is a tertiary care center as well as a public sector teaching and training institute located in central India. Data were collected by interview technique using a predesigned and pretested questionnaire. Data were analyzed using the statistical software Epi Info version 7.2.5 (CDC, Atlanta, Georgia). Mean and standard deviations were calculated for continuous variables and the chi-square test was applied for categorical variables.

Results

Of the 612 study subjects, 409 (66.8%) belonged to the clinical disciplines and 203 (33.2%) were from nonclinical and paraclinical disciplines. A total of 66 (32.5%) subjects from the paraclinical and nonclinical sides had experienced pregnancy during residency, whereas only 54 (13.2%) from the clinical side were pregnant during residency. Positive influences for pregnancy during residency were concerns about age and fertility, pressure from in-laws and parents, desire for family and pregnancy, etc., all with a mean score of 3.5 and above on a five-point Likert scale. Tight schedules, availability of childcare arrangements, support from faculty and other residents, etc. were relatively negative influences with a mean score of less than 3.5. Around 66% of those from nonclinical and paraclinical groups had conceived before 26 years of their age, whereas only 30% of residents from clinical departments had experienced pregnancy before that age. Thus, the age at conception was relatively lower in residents from nonclinical and paraclinical disciplines as compared to their counterparts from clinical disciplines, and this difference was found to be statistically significant (p < 0.001). The complications during pregnancy were more in clinical residents than in those from the nonclinical and paraclinical side.

Conclusions

This study concludes that concerns about age and fertility, pressure from in-laws and parents, desire for family and pregnancy, and enjoying children are relatively positive influences on the occurrence of pregnancy, whereas tight schedule, availability of childcare arrangements, support from faculty and other residents, and timing professionally are relatively negative influences.

## Introduction

Recent decades have witnessed a marked influx of females in the medical profession across the globe, with India being no exception [1-5]. The integration of family and career poses a significant problem for women in the medical profession. It may also be a determinant in the choice of specialty and type of practice. Whether or not to have children and if so when, constitute major decisions for women in medicine [6]. Balancing residency program demands with burgeoning family issues has always been a dilemma for medical residents.

A female medico may choose to conceive during various phases of her life, but each obviously has its own positive and negative considerations. During pre-residency days, they have more time with more flexible schedules and the woman is in the prime of her reproductive span, but in contrast, if she opts for pregnancy, finances and future childcare arrangements can be bothersome issues [2]. During residency, a woman is still at the prime of her reproductive years with the finances improved, but long working hours, inflexible duty schedules, lack of support system, difficulty in finding child care, and possible disruption of the educational process are negative considerations [2,6]. After residency, though the working hours may be more flexible and finances improved, possible concerns of infertility and difficulty in arranging maternity leave depending upon her specialty and practice pose a problem. Also, stress factors related to child care and encroachment upon professional lifestyle are worrisome. Of concern for some is the decreased fertility with increasing age and the guilt associated with the belief that pregnancy would have been easily possible had she attempted conception at an earlier age [6].

The dilemma between biological and professional clocks is a particularly important issue that several female physicians face, one which may have critical implications in terms of professional advancement and potential career satisfaction [2,7]. Despite this fact, delayed child-bearing appears to be common among female medicos [7].

Although residency occurs at an age when many females want to have children, most residency programs have no specific policies regarding maternity leave [8,9]. Many pregnant residents perceive resentment of the pregnancy from colleagues and faculty members [2,7,10]. Residents who have children during training encounter many difficulties like sleep deprivation and tiredness, on-call responsibilities with long and unpredictable work hours, difficulties with child care, and breastfeeding problems [10]. Lack of support and sometimes hostility from partners, program administrators, teachers, and other residents have also been reported [2,6,10]. All these factors, including the rigorous demands during residency and the knowledge that sick leave has an adverse impact on the workload of colleagues, act as a deterrent because of the negative career implications and the resultant maternal discrimination. This in turn may influence the decision to marry and conceive [11].

The fact that becoming pregnant has widespread implications beyond the individual to the residency program and its other members also dissuades these women from becoming pregnant [4]. Most women end up taking time off from their careers to accommodate a child, and this time is usually leave without pay and at the cost of their career advancement [12]. The literature reports that residents often struggle with the conflicting identities and roles of mothers, students, and physicians [10,13,14]. Planning a pregnancy at a proper time during the resident training schedule will go a long way in minimizing disruption to the resident’s education and the stress perceived [15]. It is essential that these issues be analyzed for the smooth operation of the residency program and the most effective training of physicians. Yet there is a dearth of literature on this topic, with very few Indian studies critically examining these concerns. With this backdrop, the present study was carried out to assess perceptions and experiences of female medicos regarding pregnancy during residency.

## Materials and methods

Study design and setting

The present descriptive cross-sectional study was carried out at Government Medical College and Hospital, Nagpur, which is a tertiary care center as well as a public sector teaching and training institute located in central India.

Study population

This study was carried out on female medical graduates across various departments of the study setting, who were currently attending or had recently completed the residency program.

Sampling and sample size

As there was no literature from India documenting the occurrence of pregnancy during residency, the authors calculated the sample size based on findings of the pilot study carried out on 50 female medicos, which reported the proportion of pregnancy during residency to be 11 (22%). Considering a relative precision of 15% and desired confidence level of 95%, the estimated sample size was calculated to be 605. However, the actual sample size as covered by the convenience sampling technique was 612.

Ethical considerations

Approval from the Institutional Ethics Committee of Government Medical College, Nagpur was obtained as per letter number 3590 (dated: 25/1/2022). Permission from the institutional head was taken. Written informed consent was taken from the study subjects after apprising them of the nature and purpose of the study.

Data collection and study tool

The study subjects being of comparable age, socioeconomic status, and educational background were grouped into two categories: those from clinical branches (medicine, surgery, obstetrics and gynecology, etc.) and those from non- and paraclinical branches (anatomy, physiology, pharmacology, microbiology, etc.). This categorization was preferred because residents from clinical branches have similar physical demands during residency and their program offers little or no flexibility for working hours or time off. The study subjects were interviewed in person using a predesigned and pretested questionnaire (Appendix).

The questionnaire was constructed by the lead researcher and was reviewed by a panel of experts for content validity and reliability. It was piloted on 10 postgraduate students from various departments and necessary modifications were made accordingly. These subjects were then excluded from the final analysis. The questionnaire comprised three parts: the first part included information regarding the socio-demographic characteristics of the study subjects. In the second part, respondents were asked to rank 20 factors that may influence the decision to have a child during residency on a five-point Likert scale with a score of 5 representing a strongly positive influence and a score of 1 representing a strongly negative influence for becoming pregnant whereas a score of 3 indicated a neutral influence. A score of more or less than 3 indicated a positive or negative influence, respectively.

In the third part of the questionnaire, those respondents who were pregnant were further enquired about whether the pregnancy was planned or not and what was the reason for choosing that particular time for the pregnancy. Both contraception user failures and method failures were regarded as unplanned while considering the issue of planned versus unplanned pregnancy. Other questions dealt with complications during pregnancy, the outcome of pregnancy, support during the pregnancy and afterward, childcare arrangements, and breastfeeding practices.

Data analysis plan

Data were analyzed using the statistical software Epi Info version 7.2.5 (CDC, Atlanta, Georgia). Mean and standard deviations were calculated for continuous variables and the chi-square test was applied for categorical variables.

## Results

Of the 612 study subjects, 409 (66.8%) belonged to the clinical disciplines and 203 (33.2%) were from nonclinical and paraclinical disciplines. A total of 66 (32.5%) subjects from the paraclinical and nonclinical sides had experienced pregnancy during residency, whereas only 54 (13.2%) from the clinical side were pregnant during residency. This difference was found to be statistically significant (p < 0.001). Of the respondents, 381 (62.3%) chose not to marry to avoid conflict between career and family obligations (Table 1).

**Table 1:** Marital and pregnancy status in study subjects Chi-square = 32.0894, Df = 1, p = <0.00001.

Marital and pregnancy status	Clinical		Non/paraclinical		Total	
	Number	Percentage	Number	Percentage	Number	Percentage
Unmarried	295	72.1	86	42.4	381	62.3
Married but not pregnant	60	14.7	51	25.1	111	18.1
Married and pregnant	54	13.2	66	32.5	120	19.6
Total	409	100	203	100	612	100

To assess the impact of various factors on the decision to have a pregnancy during residency, the mean score for each of the 20 listed factors was calculated. The residents’ general perception of these influences did not vary significantly among the two categories. For ease of understanding and clarity, they have been regrouped in Table 2 by the relative impact they appeared to have. Concerns about age and fertility, pressure from in-laws and parents, desire for family and pregnancy, and enjoying children were viewed as relatively positive influences to become pregnant. On the other hand, tight schedules, availability of childcare arrangements, support from faculty and other residents, and timing professionally were seen as relatively negative influences.

**Table 2:** Relative ranking of factors involved in deciding to begin or continue a pregnancy during residency (N = 612) Values represent an average ranking, based on scores of 1 (strongly negative) to 5 (strongly positive).

Positive influences		
1.	Concern about her age	4.80
2.	Pressure from in-laws	4.75
3.	Concern about decreasing fertility	4.60
4.	Desire for family	4.25
5.	Pressure from parents	4.19
6.	Enjoys and loves children	3.90
7.	Desires for pregnancy	3.75
Neutral influences		
8.	Fears of medical risks	3.44
9.	Proper time personally to conceive	3.30
10.	Financial feasibility	3.24
11.	Pressure from partner	3.18
12.	Pressure from friends in other fields	3.06
13.	Wants to get childbearing over	3.02
14.	Partner’s age	2.60
Negative influences		
15.	Support from faculty	2.48
16.	Support from male co-residents	2.24
17.	Support from female co-residents	1.96
18.	Proper time professionally to conceive	1.60
19.	Availability of childcare arrangements	1.23
20.	Tight schedule vigorous work	1.12

Table 3 depicts the age at conception of residents who were pregnant during residency. The age at conception was relatively lower in residents from nonclinical and paraclinical disciplines as compared to their counterparts from clinical disciplines and this difference was found to be statistically significant (p < 0.001). Around 66% of the nonclinical and paraclinical group had conceived before the age of 26 years and approximately 70% in the clinical group conceived after the age of 26 years. Most of them were about to complete their residency around the expected dates and hence had planned their pregnancies accordingly.

**Table 3:** Distribution of study subjects as per age at conception Chi-square = 16.3424, Df = 3, p = 0.000965.

Age at pregnancy	Clinical		Non/paraclinical		Total	
	Number	Percentage	Number	Percentage	Number	Percentage
22-24	04	7.4	12	18.1	16	13.3
24-26	12	22.2	32	48.5	44	36.7
26-28	30	55.6	17	25.8	47	39.2
>28	08	14.8	5	7.6	13	10.8
Total	54	100	66	100	120	100

Out of the 54 total pregnancies from clinical disciplines, 21 (38.9%) were planned pregnancies, but only 22 (40.7%) of the 54 actually desired it. On the other hand, out of the 66 pregnancies in the nonclinical and paraclinical disciplines, 46 (69.7%) had planned the pregnancy and 48 (72.7%) of the 66 pregnancies were actually desired ones. There were study subjects who desired pregnancy and planned for it, there were others who did not desire it and thus decided against it. Unplanned pregnancies accounted for about 53 (44.2%) of the reported pregnancies (Table 4).

**Table 4:** Planned versus unplanned pregnancies

Pregnancy	Clinical (n = 54)		Non/paraclinical (n = 66)		Total	
	Number	Percentage	Number	Percentage	Number	Percentage
Planned	21	38.9	46	69.7	67	55.8
Unplanned	33	61.1	20	30.3	53	44.2
Unplanned pregnancies						
1. User failure	30	90.9	19	95	49	92.5
2. Method failure	3	9.1	1	5	4	7.5
Desired	22	40.7	48	72.7	70	58.3
Undesired	32	59.3	18	27.3	50	41.7

**Figure 1:**
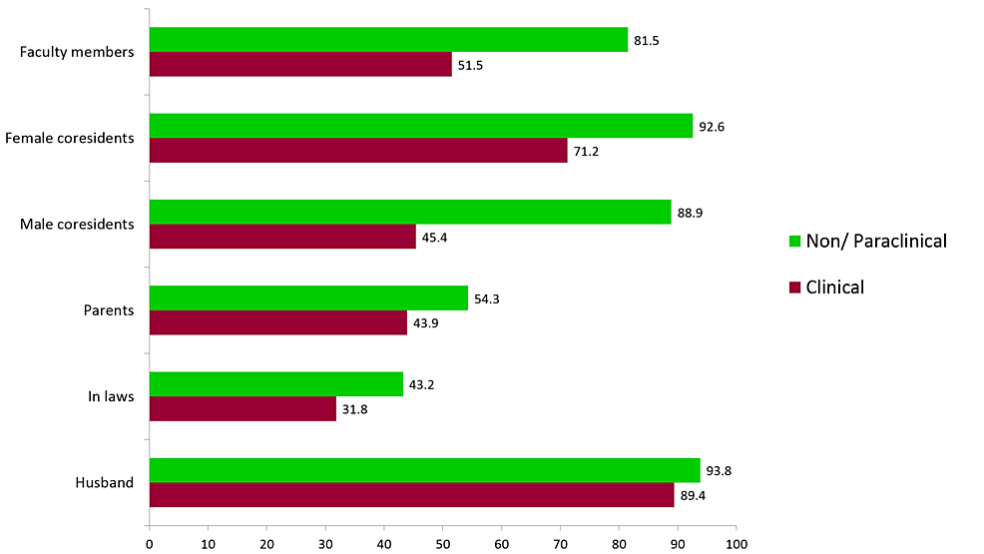
Support received during pregnancy

Study subjects were also enquired regarding the support they received during pregnancy. Though the majority (93.8%) reported that they were well supported by their husbands, the figures for in-laws and parents are not quite satisfactory. This may be because of the ever-increasing trend of nuclear families and also because many of them were staying in hostels. As far as support from faculty members and co-residents was concerned, those from the nonclinical and paraclinical sides reported greater support than those from the clinical sides (Figure 1).

**Figure 2:**
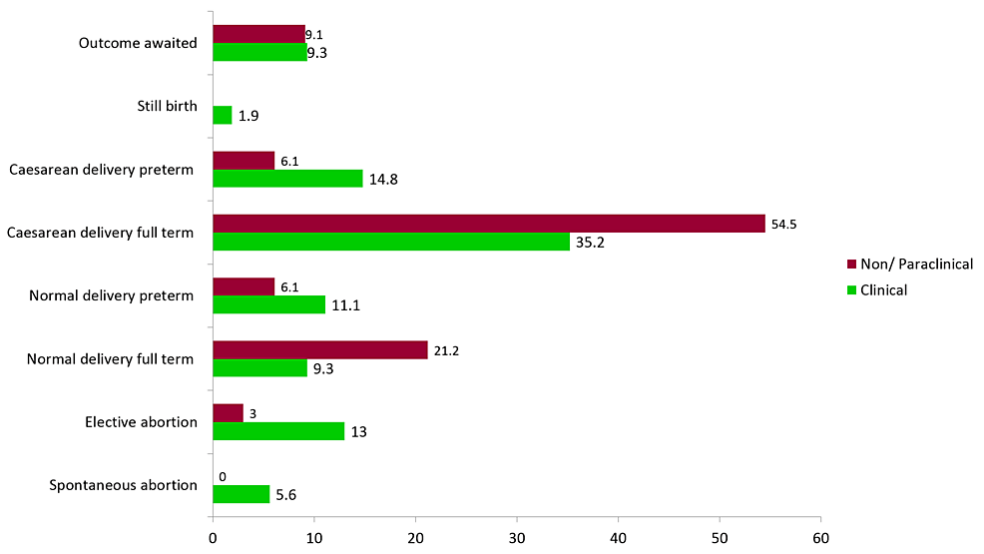
Outcome of pregnancies

The outcomes of pregnancies are seen in Figure 2. Although the number of abortions, both spontaneous and elective, was more in clinical subjects, we cannot comment much on it because of the small sample size.

**Figure 3:**
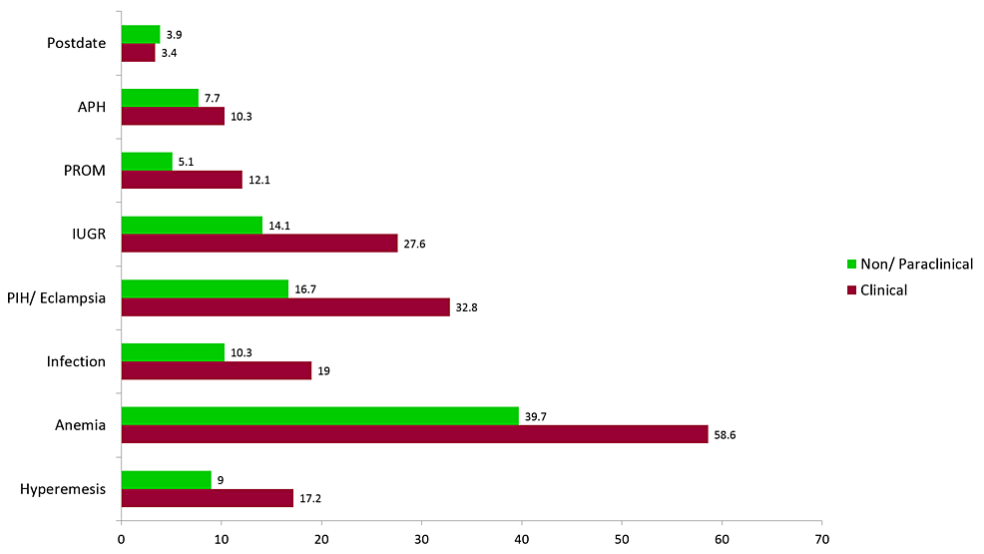
Complications during pregnancy APH: antepartum hemorrhage; PROM: premature rupture of membranes; IUGR: intrauterine growth restriction; PIH: pregnancy-induced hypertension.

Regarding complications during pregnancy, they were, in general, more in clinical residents than in those from nonclinical and paraclinical sides (Figure 3).

As far as childcare arrangements are concerned, substitute child care was provided by one of the family members in 45 (37.5%) respondents and 78 (65%) had also employed a paid caretaker. Some of them kept their child in daycare centers. Eighty-nine (74.2%) were not satisfied with the quality of child care and had a feeling of guilt. Study subjects were also enquired about breastfeeding practices. It was found that pre-lacteal feeds were given in 38 (31.6%). Only 39 (32.5%) exclusively breastfed for the first six months. The most common reason for the introduction of artificial feeds was busy work schedules and inconvenience. Expressed breast milk was given by only 15 (12.5%) of the study subjects. Thirty-one (25.8%) reported that their husbands helped them with household chores and in the case of 50 (41.7%) respondents, they helped in looking after the child.

## Discussion

The phenomenon of the mother having the sole responsibility for child rearing with no help from extended family is becoming quite common due to the increase in nuclear family trends [16]. Added to this, the recent trend toward more and more people opting for post-graduation because of the general bend toward specialization highlights the importance and relevance of this study.

The impact of childbearing on a medical career cannot be underestimated. Medicine and motherhood are seen by society as full-time but separate careers. A physician is always supposed to be available to her patients just as a mother should always be available to her children [11,13]. Each role has certain uncertainties, rewards, and demands, many mutually exclusive and draining on women’s time and energy [13].

The decision to have a pregnancy during residency depends upon a number of factors. Positive influences on the occurrence of pregnancy as reported by the respondents in this study include concerns about age and fertility, pressure from in-laws and parents, desire for family and pregnancy, and enjoying children. Similar findings were noted by other authors also [2,10,17].

The main stressors of pregnancy during residency as cited by the residents in this study are tight schedules, availability of childcare arrangements as well as support from faculty and other residents, which is similar to the findings of other studies [2,10,18-20]. As more women pursue careers in medicine, the number of female medical residents who become pregnant each year is also increasing. This highlights the need for medical institutions to develop policies and support systems that ensure pregnant residents are not disadvantaged in their training or career advancement. Despite the enormous increase in the number of women entering the health workforce, the healthcare workplace is not fully adapted to accommodate the physical demands of pregnancy or motherhood.

Almost two-thirds of respondents chose not to marry as a method of avoiding conflict between career and family obligations. A significantly greater number of residents from the non- and paraclinical specialties were married and had experienced pregnancy as compared to those from clinical specialties. The age of marriage and pregnancy was significantly higher in residents from the clinical side. Residents from clinical specialties are overburdened with their packed schedule and hospital duties allowing no time for family, let alone the added responsibility of child-rearing. Differential pregnancy rates in clinical and nonclinical specialties could also be due to negative attitudes from colleagues and seniors. Similar findings have been suggested in other studies [18,19].

Irrespective of the specialty and the phase of medical education, getting pregnant at any stage of the training for a female medico has its own advantages and disadvantages [6]. The combination of five-and-a-half years of under graduation and internship and at least three additional years of post-graduation training leads to a potential increase in childbearing age. The savant woman is cognizant of the fact that a sizeable proportion of women may suffer from relative or absolute infertility and that the longer the pregnancy is delayed, the lower is the conception rate. Still, female medicos are reluctant to have pregnancy during residency because of consequent interference of study, isolation from peers, financial demands, lack of support system, and concern that their image as serious professionals would be compromised.

Some study subjects desired pregnancy and accordingly planned for it; however, there were some who did not desire it and thus decided against it. In the present study, unplanned pregnancies constituted about half of the reported pregnancies. A pregnancy that is not timed correctly may negatively impact both education and personal life, a fact that undoubtedly accentuates the importance of a planned pregnancy. It was observed that most of the respondents with unplanned pregnancies actually desired to have children as intensely as those who had planned their pregnancies, but for some reason or other were unable to time it properly, professionally or personally.

The key supporters during pregnancy were husbands and female residents as reported by more than three-fourths of the study participants from nonclinical specialties and almost half of the participants from clinical specialties in the present study. However, Sugimoto et al. noted that colleagues, preceptors, and residency program affiliates were generally supportive of pregnant residents [21].

Medical residents are highly educated yet they spend long hours during both day and night in work that includes prolonged standing and great emotional stress.

It was observed that the occurrence of pregnancy-related complications and adverse outcomes was more in residents from clinical specialties as compared to their counterparts from nonclinical and paraclinical sides. Prenatal advice usually offered to normal pregnant women includes “an adequate amount of rest and avoid becoming overtired.” This advice and usual suggestions of frequent meals, reduced activity, and rest periods during the working day are difficult to satisfy during residency or practice, particularly by those in clinical fields. Another reason for a higher number of abortion complications in residents from the clinical side could be the higher age both at marriage and conception in them.

Strengths and limitations

The present study has multiple strengths, prominent amongst them being its unique nature and the fact that despite being an important issue, it has remained largely unexplored with very few studies globally and hardly any from India. Due care taken to customize the questionnaire and validate it so as to fulfill the study objectives is another plus point of the current study. The sample covered for the current study is reasonably large and representative. As the study covered some sensitive issues, the possibility of social desirability, recall, and other similar biases cannot be ruled out.

## Conclusions

This study concludes that concerns about age and fertility, pressure from in-laws and parents, desire for family and pregnancy, and enjoying children are relatively positive influences on the occurrence of pregnancy, whereas tight schedule, availability of childcare arrangements, support from faculty and other residents, and timing professionally are relatively negative influences. Occurrence of complications and adverse outcomes are higher in respondents from clinical specialties as compared to those from non- and paraclinical specialties. This indicates that although the medical profession is one of the most cherished professions in our country, the medical workplace has been slow to attune to childbearing and parenting. Balancing a medical career and motherhood poses significant challenges for women, and requires targeted support and further research. Women need access to reliable information to make informed decisions about the timing of pregnancy, so more of such studies should be carried out on a large scale preferably at the national level.

It is important to recognize that motherhood is not the only issue nor is medicine. For any working woman, there is a dual problem of career and family, important issues being whose career takes precedence and who takes responsibility of children. The social institution of medicine should take a leadership role. Women physicians (and their spouses) who successfully cope with the career-family dilemma can serve as role models to millions of Indian families in which the mother is employed outside the home. To better accommodate an increasing percentage of women, the structure of residency programs may need to be modified. Additionally, childcare support systems should be developed for both residents and faculty, and facilities such as maternity leave and breastfeeding support should be provided to ensure that psychological, social, and physical obstacles do not compromise the attraction of a medical career for women.

## Appendices

Appendix

**Table 5:** Questionnaire for interview APH: antepartum hemorrhage; PROM: premature rupture of membranes; IUGR: intrauterine growth restriction; PIH: pregnancy-induced hypertension.

Section A: General information	
Variable	Response
Name	
Age in years	
Current cadre	Faculty/junior resident/senior resident
Department	Clinical/paraclinical/nonclinical
Marital status	Unmarried/married

Section B: Factors influencing the decision to become pregnant					
Rank the following factors on a five-point scale with a score of 5 representing strongly positive influence and a score of 1 indicating a strongly negative influence for becoming pregnant					
Factor	1	2	3	4	5
Pressure from partner					
Support from male co-residents					
Enjoys and loves children					
Concern about decreasing fertility					
Tight schedule vigorous work					
Proper time personally to conceive					
Concern about her age					
Availability of childcare arrangements					
Pressure from parents					
Pressure from in-laws					
Wants to get childbearing over					
Desires for pregnancy					
Partner’s age					
Desire for family					
Fears of medical risks					
Financial feasibility					
Proper time professionally to conceive					
Pressure from friends in other fields					
Support from faculty					
Support from female co-residents					

Section C: Questions related to pregnancy	
Whether you experienced pregnancy during residency?	Yes/No
Answer the following questions only if the answer to the above question is yes	
Age at pregnancy	
Whether the pregnancy was planned/unplanned?	
If unplanned, user failure/method failure	
Whether the pregnancy was desired/undesired?	
Source of support received during pregnancy	Husband/parents/in-laws/male co-residents/female co-residents/faculty members/others, specify
Outcome of pregnancy	Spontaneous abortion/elective abortion/normal delivery full-term/normal delivery pre-term/cesarean delivery full-term/cesarean delivery preterm/stillbirth/outcome awaited
Complications during pregnancy	Hyperemesis/anemia/infection/PIH and eclampsia/IUGR/APH/PROM/post date
Childcare arrangements	Family members/paid caretakers/daycare center
Did your husband help you with the household chores?	Yes/No
Satisfied with the quality of childcare	Yes/No
Breast-feeding practices	
Prelacteal feeds given	Yes/No
Exclusive breastfeeding for 6 months	Yes/No
Was expressed milk given?	Yes/No
Whether given artificial feeds	Yes/No
If yes, reasons for giving artificial feeds	
